# Biological and clinical characteristics of *ETV6*::*RUNX1*‐like ALL

**DOI:** 10.1002/hem3.70342

**Published:** 2026-04-17

**Authors:** Marketa Zaliova, Dagmar Schinnerl, Judith M. Boer, Aurélie Caye‐Eude, Jacqueline Rehn, Claire Schwab, Chloé Arfeuille, Andishe Attarbaschi, Anke Katharina Bergmann, Karel Fiser, Hester A. de Groot‐Kruseman, Sabrina Haslinger, Andrea Inthal, Iveta Janotova, Lennart Lenk, Margarita Maurer‐Granofszky, Karin Nebral, Fiona Poyer, Lucie Sramkova, Jan Stary, Marion Strullu, Jan Stuchly, Rosemary Sutton, Martina Vaskova, Lucie Winkowska, Gunnar Cario, Hélène Cavé, Monique L. den Boer, Christine Harrison, Sabine Strehl, Deborah White, Jan Trka, Jan Zuna

**Affiliations:** ^1^ Department of Paediatric Haematology and Oncology, Second Faculty of Medicine Charles University Prague Czech Republic; ^2^ CLIP (Childhood Leukaemia Investigation Prague) Prague Czech Republic; ^3^ University Hospital Motol Prague Czech Republic; ^4^ St. Anna Children's Cancer Research Institute (CCRI) Vienna Austria; ^5^ Princess Máxima Center for Pediatric Oncology Utrecht The Netherlands; ^6^ Department of Genetics Robert Debré Hospital and Université Paris Cité Paris France; ^7^ South Australian Health & Medical Research Institute (SAHMRI) Adelaide South Australia Australia; ^8^ University of Adelaide, Faculty of Health and Medical Science Adelaide South Australia Australia; ^9^ Leukaemia Research Cytogenetics Group, Wolfson Childhood Cancer Research Centre, Translational and Clinical Research Institute Newcastle University Newcastle upon Tyne United Kingdom; ^10^ St. Anna Children's Hospital Medical University of Vienna Vienna Austria; ^11^ Clinical Genetics and Genomic Medicine University Hospital Würzburg Würzburg Germany; ^12^ Hannover Medical School Hannover Germany; ^13^ Department of Bioinformatics, Second Faculty of Medicine Charles University Prague Czech Republic; ^14^ Labdia Labordiagnostik Vienna Austria; ^15^ Department of Pediatrics Christian‐Albrechts‐University Kiel and University Medical Center Kiel Germany; ^16^ Department of Pediatric Hemato‐oncology Robert Debré Hospital and Université Paris Cité Paris France; ^17^ Children's Cancer Institute Australia for Medical Research University of New South Wales Sydney New South Wales Australia; ^18^ Australasian Leukaemia & Lymphoma Group (ALLG) Richmond Victoria Australia; ^19^ The Australian & New Zealand Children's Haematology/Oncology Group (ANZCHOG) Clayton Victoria Australia

## Abstract

*ETV6::RUNX1*‐like ALL is defined by a gene expression signature similar to that of *ETV6::RUNX1*‐positive ALL and absence of all genetic subtype‐defining aberrations, including the *ETV6::RUNX1* fusion. Within the International BFM Study Group, we assembled and analyzed a cohort of 100 patients (including 97 children) with *ETV6::RUNX1*‐like ALL. We describe their diverse genetic landscape, centered around *ETV6* aberrations with frequent *IKZF1* disruptions, as previously shown, but including various rare non‐*ETV6*/non‐*IKZF1* gene fusions, and rearrangements of *CRLF2* (*CRLF2*r). We show that *ETV6* and *IKZF1* aberrations do not occur exclusively in this subtype, which hampers its classification based solely on genomic data. We confirm our previous observation of a strong association of the CD27‐positive/CD44low‐negative immunophenotype with *ETV6*::*RUNX1*(‐like) subtype. Compared to *ETV6::RUNX1*‐positive ALL, patients with *ETV6::RUNX1*‐like ALL are younger, have higher white blood cell counts at diagnosis, and have an inferior early treatment response. While overall survival is comparable, event‐free survival is significantly lower in patients with *ETV6::RUNX1*‐like ALL, with NCI risk, early treatment response, *IKZF1* deletions, *CRLF2r,* and *JAK2* mutations having prognostic relevance. Notably, Down syndrome is highly prevalent and associated with a worse outcome in *ETV6::RUNX1*‐like ALL. In conclusion, we provide biological, demographic, and clinical characteristics of the largest *ETV6::RUNX1*‐like cohort presented to date.

## INTRODUCTION

Several subtypes have been established within B‐cell precursor acute lymphoblastic leukemia (BCP‐ALL), differing in genomic background, biology, and clinical features, including outcome. Most subtypes are defined by specific chromosomal/genetic aberrations, while the minority are defined by specific gene expression profiles (GEP), corresponding to one of the genetically defined subtypes in the absence of its defining genetic aberration.^1^ This definition applies to *ETV6::RUNX1*‐like ALL characterized by a GEP indistinguishable from ALL with the *ETV6::RUNX1* fusion gene. Pediatric studies published to date were mostly not focused on this subtype and, except for a previous study from our group,^2^ not “population‐based.” They estimate the frequency of *ETV6::RUNX1*‐like ALL to range from 1% to 4.5% within BCP‐ALL.^1^, ^2^, ^3^, ^4^, ^5^, ^6^, ^7^, ^8^ Cases classified into this subtype frequently harbor aberrations involving the *ETV6* and *IKZF1* genes, yet these lesions are not present in every case.^1^, ^3^, ^4^, ^6^ Limited data are available to assess the prognostic relevance of *ETV6::RUNX1*‐like ALL. While *ETV6::RUNX1*‐positive ALL represents one of the most favorable subtypes of pediatric BCP‐ALL,^9^, ^10^, ^11^, ^12^ the prognosis of *ETV6::RUNX1*‐like ALL is considered to be intermediate, based on smaller studies including less than 20 *ETV6::RUNX1*‐like patients—better than high‐risk ALL subtypes yet inferior to *ETV6::RUNX1*‐positive ALL.^1^, ^5^, ^6^

We conducted a retrospective study within the International BFM Study Group (IBM‐SG), focusing on the genomic, demographic, and clinical aspects of *ETV6::RUNX1*‐like ALL, particularly in children. We collected data from 100 patients (including 97 children) with *ETV6::RUNX1*‐like BCP‐ALL, representing the largest studied cohort with this ALL subtype so far.

## MATERIALS AND METHODS

### Patients and samples

In total, 2027 samples with sufficient leukemic cells from 1709 children and 318 adults diagnosed with BCP‐ALL in Australia (AUS; *n* = 567), Austria (AUT; *n* = 317), Czech Republic (CZE; *n* = 323), France (FRA; *n* = 244), Germany (GER; *n* = 43), United Kingdom (UK, *n* = 28), and Netherlands (NLD; *n* = 505) were included in whole transcriptome sequencing (WTS) based gene expression studies.Patients were selected based on the availability of existing WTS data or material suitable for WTS. A subgroup of 84 patients was further included based on the presence of *ETV6* gene deletions (*ETV6*del) in the absence of routinely screened genetic aberrations. The Czech cohort included 170 children representing 96% of all Czech patients diagnosed consecutively from December 2010 to October 2021 and negative for all routinely screened genetic aberrations (high hyperdiploidy, hypodiploidy, *ETV6::RUNX1*, *TCF3::PBX1*, *KMT2A*r, *BCR::ABL1*). A detailed description of the cohorts is provided in Supporting Information S1: Table 1.

Demographic, clinical, and genetic data were obtained from 100 patients classified as *ETV6::RUNX1*‐like. Immunophenotyping, cytogenetics, and diagnostic genetic investigations were performed as part of the standard laboratory work‐up.

**Figure 1:**
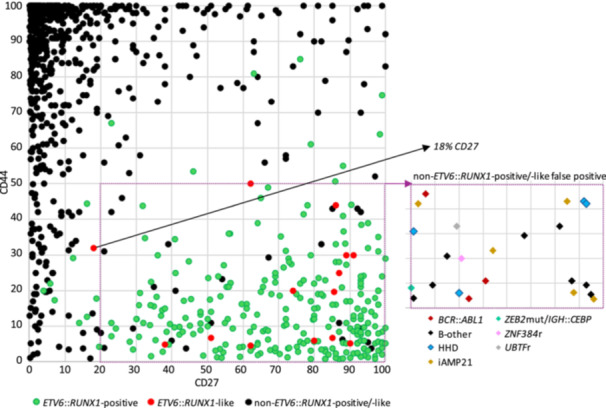
**Expression pattern of CD27/CD44**. Surface expression of CD27 and CD44 was measured by flow cytometry in 978 diagnostic BCP‐ALL samples. The sample set includes 263 *ETV6*::*RUNX1*‐positive, 14 *ETV6*::*RUNX1*‐like, and 701 BCP‐ALL cases classified by genetic and gene expression profiling as non‐*ETV6*::*RUNX1*‐positive/‐like. The subtypes of the 23 cases that would be falsely predicted as *ETV6*::*RUNX1*‐positive/‐like using cut‐offs of ≥20% for CD27 and ≤50% for CD44 are detailed in the graph snippet on the right. *X*‐axis (*Y*‐axis) represents the percentage of leukemic cells expressing CD27 (CD44).

Demographic, clinical, and genetic data from additional patients diagnosed and treated in the Czech Republic from December 2010 to October 2021 were included in comparative analyses (Supporting Information S1: Figure 1).

All diagnostic and research procedures were performed according to approval of local Institutional review boards and in accordance with the Declaration of Helsinki. Written informed consent was provided by patients/legal guardians.

### *ETV6::RUNX1*‐like subtype classification

WTS data sets were collected and analyzed in five centers (AUS, AUT, CZE, FRA, and NLD; samples from GER and UK were analyzed in CZE) using various locally established bioinformatic pipelines. Each center performed gene expression and clustering analyses, including hierarchical clustering analysis or dimensionality reduction using the t‐Distributed Stochastic Neighbor Embedding (tSNE) method. Samples lacking all subtype‐defining genetic aberrations, which co‐clustered with *ETV6::RUNX1*‐positive ALL in unsupervised clustering analysis, were classified as *ETV6::RUNX1*‐like. In line with the most extensive study to date on the classification of BCP‐ALL^1^, other genetic aberrations, including *PAX5*r, *CRLF2*r, and kinase or cytokine receptor gene fusions other than *BCR::ABL1,* were not considered as subtype‐defining, thus did not preclude their classification into the *ETV6::RUNX1*‐like subtype.

### Evaluation of CD27/CD44 markers expression

Expression of CD27/CD44 was measured by flow cytometry, as previously described,^13^, ^14^ in diagnostic BCP‐ALL samples of patients with sufficient material for analysis, who were diagnosed in the Czech Republic from May 2015 to November 2021. These data were pooled with data from our published study,^14^ which included patients diagnosed from March 2003 to April 2015.

### Genetic characterization of *ETV6::RUNX1*‐like BCP‐ALL

Data from WTS were used for the identification of fusions, other structural aberrations, and single‐nucleotide variants (SNV) and insertions/deletions (indels) in selected genes (Supporting Information S2: Table 2).

Genomic profiling using SNP‐arrays, whole exome sequencing (WES) of diagnostic samples (or diagnostic and nontumor sample pairs), and targeted DNA sequencing of selected genes (Supporting Information S2: Table 2) were performed in 57, 13, and 22 patients, respectively.

In cases where DNA‐level data (WES or targeted NGS) were not available, WTS was used for variant calling. Although this approach may have slightly lower sensitivity, the complete concordance observed in cases with both data types suggests that any missed variants would be rare.

Copy number aberrations of *ETV6*, *CDKN2A*, *CDKN2B*, *PAX5*, *IKZF1*, *EBF1*, *BTG1*, *RB1*, and of the PAR1 region were analyzed by Multiplex Ligation‐dependent Probe Amplification (SALSA MLPA Probemix P335 ALL‐IKZF1, MRC Holland) in 67 patients. In total, 91/100 patient samples were analyzed by SNP‐array and/or MLPA. Cytogenetic data were available from 86 patients. All results (Supporting Information S2: Tables 3–6) were integrated to define the genetic background of *ETV6::RUNX1*‐like ALL.

### Statistical analyses

Overall survival (OS) was calculated as the time from diagnosis to death. Event‐free survival (EFS) was defined as the time from diagnosis to relapse, a second tumor, or death; induction failure was considered an event at Day 0. Time was censored at the date of the last patient contact in the absence of an event. Relapse‐free survival (RFS) was defined as the time from diagnosis to relapse, censoring at other events. Survival rates were calculated according to Kaplan–Meier and compared by the log‐rank test. Univariate Cox regression models were used to determine hazard ratios (HR).

For multivariate analysis, Cox proportional hazards models were constructed, and the model selection was performed using the Akaike Information Criterion (AIC). Results from the best model (based on AIC) are presented. Due to partially missing data for some variables in varying subsets of patients, the analysis of mutual independence of their prognostic impact had to be performed via two separate multivariate analyses using partially overlapping variable sets to avoid losing a significant proportion of data, which would skew the results. Other comparisons were performed using the Mann‐Whitney *U* test or Fisher's exact tests as appropriate. All probability (P) values were two‐sided; P < 0.05 were considered statistically significant.

## RESULTS

### Identification of *ETV6::RUNX1*‐like ALL

**Figure 2:**
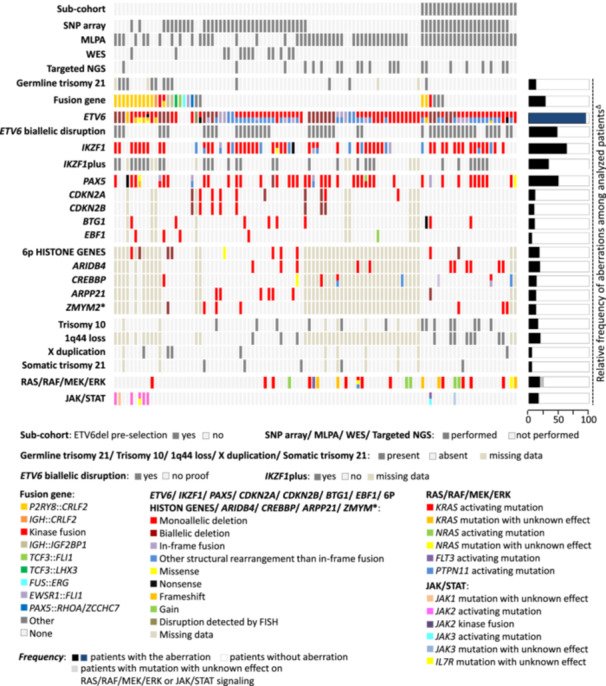
**Genetic background of** ***ETV6*** **::** ***RUNX1*** **‐like BCP‐ALL**. Selected genetic findings in patients with *ETV6*::*RUNX1*‐like BCP‐ALL (*n* = 100). The figure depicts methods used for genomic profiling of 100 patients with *ETV6*::*RUNX1*‐like ALL (of note, WTS was performed in all patients), and aberrations of 20 genes/regions, which were affected in ≥5 out of the analyzed patients. Deletions on chromosomes 12 and 7 (including monosomies 12 and 7) affecting *ETV6* and *IKZF1*, respectively, are shown as *ETV6*/*IKZF1* deletions. Aberrations of *KRAS*, *NRAS*, and *JAK2* genes, which are among these 20 genes/regions, are included in lanes showing aberrations of additional (less frequent) RAS/RAF/MEK/ERK and JAK/STAT signaling pathway genes. These mutations are labeled as activating when the mutation activates downstream signaling according to published studies, or unknown when the impact on downstream signaling has not been studied. ^∆^, *ETV6* aberration frequency was calculated in 76 patients not preselected for *ETV6* deletions. *, includes deletions affecting *ZMYM2* gene or adjacent region (chr13:20486123‐20509959); 1q44, chr1:244483375‐249200918.

To identify *ETV6::RUNX1*‐like cases, gene expression‐based clustering analyses were performed independently in five collaborating centers using non‐overlapping sub‐cohorts of 244–567 patients each (2027 BCP‐ALL patients in total). Each sub‐cohort included representatives of 14–17 genetically defined BCP‐ALL subtypes^1^ (Supporting Information S1: Table 1). A total of 101 ALL cases (from 98 children and three adults) were classified as *ETV6::RUNX1*‐like, based on established criteria: co‐clustering with *ETV6::RUNX1*‐positive ALL and absence of all 17 genetic subtype‐defining aberrations^1^ [*ETV6::RUNX1*, *BCR::ABL1*, *TCF3::PBX1*, *TCF3::HLF*, *KMT2A* rearrangements (*r*), high hyperdiploidy (>50 chromosomes; HHD), hypodiploidy (<44 chromosomes), intrachromosomal amplification of chromosome 21 (iAMP21), *DUX4*r, *ZNF384*r, *MEF2D*r, *NUTM1*r, *PAX5* P80R, *IKZF1* N159Y, *UBTF::ATXN7L3*, *BCL2*r/*MYC*r, *ZEB2* H1038R/*IGH::CEBP*] (Supporting Information S1: Figure 2).

One hundred patients with *ETV6::RUNX1*‐like ALL were further analyzed after excluding one child with missing clinical data.

### Prediction of *ETV6::RUNX1*‐like subtype by immunophenotyping

Unlike other subtypes, *ETV6::RUNX1*‐positive/‐like BCP‐ALL typically express the CD27 surface antigen but do not or only weakly express CD44, making the CD27/CD44 expression pattern a useful subtype predictor.^13^, ^14^ We analyzed the association of the CD27^pos^/CD44^low‐neg^ immunophenotype with the *ETV6::RUNX1*‐positive/‐like subtypes in a cohort of 978 patients, including data from 495 patients from our previous study^14^ and 483 patients newly diagnosed in the Czech Republic between 2015 and 2021, excluding patients with incomplete subtype classification. Building on the qualitative description in our previous paper, for this study, we used cut‐offs of ≥20% for CD27 and ≤50% for CD44. With these, we achieved 96.7% specificity for both and 93.2% and 92.9% sensitivity for predicting *ETV6::RUNX1*‐positive and *ETV6::RUNX1*‐like BCP‐ALL cases, respectively. The only *ETV6::RUNX1*‐like case missed by these thresholds had expected CD44 expression (32%), but narrowly lower expression of CD27 (18%). Among 23 false positive cases (3% of the non‐*ETV6::RUNX1*‐positive/‐like patients), 15 belonged to six distinct genetic subtypes, and eight were negative for all subtype‐defining genetic aberrations (Figure 1).

### Genetic characterization of *ETV6::RUNX1*‐like

Genetic findings and methods employed for their detection are summarized in Figure 2 and detailed in Supporting Information S2: Tables 3–6.

**Figure 3:**
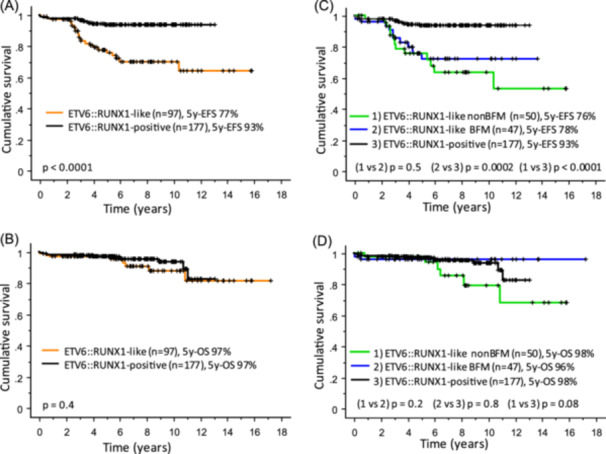
**Outcome of pediatric patients with** ***ETV6*** **::** ***RUNX1*** **‐like versus** ***ETV6*** **::** ***RUNX1*** **‐positive BCP‐ALL**. **(A**, **B)** Treatment outcomes of *ETV6*::*RUNX1*‐like (*n* = 97) and *ETV6*::*RUNX1*‐positive (*n* = 177) patients. **(C**, **D)** Treatment outcomes of *ETV6*::*RUNX1*‐like patients stratified according to the type of treatment protocol (BFM, MRD‐guided AIEOP‐BFM protocols 2000, 2009, and 2017; nonBFM, other protocols, for details see Supporting Information S2: Table 6), compared to *ETV6*::*RUNX1*‐positive (*n* = 177) patients. Censoring times are indicated by short vertical lines. EFS, event‐free survival; OS, overall survival; y, year.

*ETV6* and *IKZF1* were the only genes altered in the majority of patients (96% and 59%, respectively). *ETV6* deletions were found in 89% of cases (85 of 96 tested), and rearrangements, including those resulting from deletions, were found by WTS and/or FISH in 48% (48/100). Partner genes/regions of *ETV6* were enriched on chromosomes 12 and 7 (Supporting Information S1: Figure 3). In 13 cases, the rearrangement resulted in the formation of an in‐frame gene fusion (including *ETV6::IKZF1*, *ETV6::RUNX2*, and *KRAS::ETV6*). Twelve point‐mutations of *ETV6* were found in 10 patients, 10 of which were frameshift or nonsense. Biallelic *ETV6* alterations were evidenced in 47 patients.

*IKZF1* deletions were found in 47/91 tested patients (52%), rearrangements (including three in‐frame gene fusions) in 15/100 (15%) and SNV/indel in 2/100 tested patients (2%). Of 41 patients in whom no *IKZF1* lesions were found, 7 were not tested for *IKZF1* deletions (only WTS data were available). The *ETV6* gene was altered in all 59 patients with *IKZF1* alterations.

*PAX5*, the third most commonly affected gene, was altered in 49% of patients (45 of 92 tested): deletions were found in 39 and fusions/SNV/indel in another six patients. Out of the 47 patients with *IKZF1*del, 30 had an *IKZF1*plus genotype, the most frequent being a combination of *IKZF1* and *PAX5* deletions.

Among 84 patients intentionally included in the WTS study based on the known presence of *ETV6*del, 29% (24/84) were identified as *ETV6::RUNX1*‐like cases. The frequency of *ETV6*, *IKZF1*, and *PAX5* lesions in this sub‐cohort was comparable to the remaining *ETV6::RUNX1*‐like patients (100% vs. 95% for *ETV6* lesions, 67% vs. 62% for *IKZF1* lesions, 46% vs. 50% for *PAX5* lesions).

*CRLF2*r was found in 14 patients (14/100, 14%), 13 had *P2RY8::CRLF2* and one *IGH::CRLF2*. In addition to alterations of *ETV6*, *IKZF1*, *PAX5,* and *CRLF2*r, genetic alterations of only 16 genes/loci occurred in ≥ 5 patients each, none of them exceeding 20% frequency (deletions of *CDKN2A*, *CDKN2B*, *BTG1*, *ARID4B*, *ARPP21*, *ZMYM2* gene region or 1q44 region, gain of whole chromosomes 10, 21 or X, deletions and/or other types of aberrations of *EBF1*, 6p histone gene cluster or *CREBBP*, and mutations of *KRAS*, *NRAS* or *JAK2*; Figure 2). RAS pathway activating mutations were found in 18/100 tested patients (18%), the most frequent were *KRAS* mutations (in 12 patients). Genetic lesions activating JAK/STAT signaling were found in 16/100 (16%), 14 had *CRLF2*r (5/14 combined with *JAK2* mutations), and 2 had *PAX5*::*JAK2* (1/2 combined with *JAK3* mutation).

In addition to 15 patients with in‐frame fusions involving *ETV6* and/or *IKZF1*, and 14 patients with *CRLF2*r (co‐occurring with a *PDGFRB* fusion in 1 patient), in‐frame transcripts of various fusion genes were found in 14/100 tested patients. Ten patients harbored previously described, mostly rare fusions^1^, ^3^, ^15^, ^16^, ^17^: *PAX5::JAK2* (2), other *PAX5* fusions (three different fusions in two patients), *IGH::IG2BP1* (2), *FUS::ERG*, *EWSR1::FLI1*, *KDM2B::GATAD2B,* and *TCF3::FLI1* (in 1 patient each). Four patients had novel fusions: *TCF3::LHX3*, *CREBBP::LHX4*, *GTF2I::HOXA9,* and *DDX5::ZNF511*. In line with published studies,^16^, ^18^ *CRLF2*r and *IGH::IGF2BP1* were enriched in patients with Down syndrome (Figure 2). Interestingly, of 4 patients without *ETV6* alteration three harbored *TCF3::FLI1*, *EWSR1::FLI1,* or *FUS::ERG* fusions.

Using SNP‐array data from 167 consecutive Czech cases negative for *ETV6::RUNX1*, *BCR::ABL1*, *TCF3::PBX1*, *KMT2A*r, HHD, and hypodiploidy (including 12 consecutive *ETV6::RUNX1*‐like cases from the present study), we assessed the correlation of selected genetic lesions with the *ETV6::RUNX1*‐like phenotype. *ETV6* deletions occurred in four patients with genetically defined subtypes and in 19 patients without genetic subtype‐defining lesions (Supporting Information S1: Figure 4). Among these 19 cases, only nine (47%) had an *ETV6::RUNX1*‐like phenotype. Mutations and in‐frame fusions were rare and not exclusive to the *ETV6::RUNX1*‐like subtype. Fourteen cases had both *ETV6* and *IKZF1* lesions, eight of which (57%) had an *ETV6::RUNX1*‐like phenotype. Of the remaining six patients, two had an *ETV6::ABL1* fusion (and *BCR::ABL1*‐like phenotype), one had a *PAX5* fusion, while three cases had no subtype‐defining or other biologically determining lesion. Of note, only one of 18 patients with *CRLF2*r and no subtype‐defining genetic lesion had an *ETV6* deletion, which was the only *CRLF2*r patient with an *ETV6::RUNX1*‐like phenotype.

### Demographic and clinical features and outcome of pediatric *ETV6::RUNX1*‐like ALL

We analyzed demographic and clinical features of 97 children with *ETV6::RUNX1*‐like BCP‐ALL and compared them to 177 *ETV6::RUNX1*‐positive BCP‐ALL children diagnosed consecutively in the Czech Republic from December 2010 to October 2021 and treated according to three consecutive AIEOP‐BFM ALL protocols (2000, 2009, and 2017), which integrated minimal residual disease (MRD) into the risk stratification algorithm. Within the *ETV6::RUNX1*‐like group, we defined two subgroups: 47 patients treated according to AIEOP‐BFM (2000/2009/2017) protocols (BFM subgroup), and within them, 13 patients diagnosed consecutively in the Czech Republic (consecutive subgroup) in the same timeframe as the *ETV6::RUNX1*‐positive reference group (Table 1).

**Table 1:** Demographic and outcome features of patients with ETV6::RUNX1‐like and ETV6::RUNX1 + BCP‐ALL

	ETV6::RUNX1‐like			
	Total, *n* (%)	*non‐BFM treatment, n (%)*	*MRD‐based BFM treatment, n (%)*	ETV6::RUNX1+ *n* (%)
Total	97 (100%)	*50* (*100%)*	*47* (*100%)*	177 (100%)
Sex				
Male	50 (52%)	*24* (*48%)*	*26* (*55%)*	111 (63%)
Female	47 (48%)	*26* (*52%)*	*21* (*45%)*	66 (33%)
UN	0	*0*	*0*	0
*P‐val* a	*NS*	*NS*	*NS*	
Age (years), *n* (%)				
Median	2.9	*3*	*2.8*	4.2
Mean	3.9	*3.5*	*4.3*	4.7
<1	1 (1%)	*1* (*2%)*	*0*	0
1–(<10)	92 (95%)	*48* (*96%)*	*44* (*94%)*	169 (95.5%)
≥10	4 (4%)	*1* (*2%)*	*3* (*6%)*	8 (4.5%)
UN	0	*0*	*0*	0
*P‐val* b	<*0.0001*	*0.0001*	*0.0008*	
WBC count (10^6^/L)				
Median	18,700	*29,300*	*15,700*	6,960
Mean	35,800	*44,800*	*27,600*	15,600
<50,000	69 (77%)	*32* (*74%)*	*37* (*79%)*	162 (92%)
≥50,000	21 (23%)	*11* (*86%)*	*10* (*21%)*	15 (8%)
UN	7	*7*		0
*P‐val* b	*<0.0001*	*<0.0001*	*0.0001*	
Down syndrome				
Yes	11 (12%)	*7* (*15%)*	*4* (*9%)*	2 (1%)
No	83 (88%)	*40* (*85%*	*43* (*91%)*	175 (99%)
NA	3	*3*	*0*	0
*P‐val* a	*0.0002*	*0.0003*	*0.0187*	
EOI MRD				
Positive	56 (66%)	*24* (*62%)*	*32* (*70%)*	77 (44%)
Negative	29 (34%)	*15* (*38%)*	*14* (*30%)*	97 (56%)
UN	12	*11*	*1*	3^c^
*P‐val* a	*0.0014*	*NS* (*0.0537)*	*0.0027*	
EOI MRD				
≥1E−3	12 (14%)	*5* (*13%)*	*7* (*15%)*	3 (2%)
Positive <1E−3	44 (52%)	*19* (*49%)*	*25* (*54%)*	74 (43%)
Negative	29 (34%)	*15* (*38%)*	*14* (*30%)*	97 (56%)
UN	12	*11*	*1*	3^c^
*P‐val* a ^*∆*^	*0.0002*	*0.006*	*0.0008*	
HSCT in CR1				
Yes	2 (2%)	*0*	*2* (*4%)*	2 (1.1%)
No	88 (98%)	*81* (*100%)*	*45* (*96%)*	173 (99%)
UN	7	*7*	*0*	2^c^
Events				
Relapse	19 (20%)	*12* (*24%)*	*7* (*15%)*	7 (4.0%)
TRM	3 (3%)	*1* (*2%)*	*2* (*4%)*	3 (2%)
SM	1 (1%)	*0*	*1* (*2%)*	0
Induction failure	0	*0*	*0*	1 (0.6%)
No event	74 (76%)	*37* (*74%)*	*37* (*79%)*	166 (94%)
UN	0	*0*	*0*	0
*P‐val* a ^,^ ^d^	*0.0002*	*0.0003*	*0.0038*	

*Note*: Out of 97 patients with *ETV6*::*RUNX1*‐like ALL, 47 were treated according to BFM protocols with MRD‐based risk stratification (MRD‐based BFM treatment) and 50 were treated according to non‐BFM protocols (non‐BFM treatment) (Supporting Information S2: Table 6).

Abbreviations: *P*‐val, P values from statistical test comparing *ETV6:*:*RUNX1‐*like (sub)group with *ETV6::RUNX1*+; SM, secondary malignancy; TRM, treatment‐related mortality (1 induction death and 2 deaths in first complete remission in *ETV6::RUNX1‐*like group, 1 induction death and 2 deaths in first complete remission in *ETV6*::*RUNX1*+ group), UN, unknown.

a Fisher's exact test (two‐tailed).

b Mann–Whitney *U* test (two‐tailed); ∆, [≥1E–3] versus [positive <1E−3 and negative].

^c^ 2 patients died before EOI, 1 patient had no MRD targets.

^d^ [relapse and other event] versus [no event].

The frequency of *ETV6::RUNX1*‐positive and *ETV6::RUNX1*‐like BCP‐ALL in the Czech consecutive cohort of 705 children diagnosed from December 2010 to October 2021 was 25% and 1.8%, respectively (Supporting Information S1: Figure 1).

The male‐to‐female ratio was 52:48 in *ETV6::RUNX1*‐like ALL, while male sex tended to be more frequent (63:37) in *ETV6::RUNX1*‐positive ALL (Table 1). Patients with *ETV6::RUNX1*‐like ALL were significantly younger (median age 2.9 years, range 0.8–18.1) and had higher initial white blood cell count (WBC) (median 18.7 × 10E^9^/L, range 1–217 × 10E^9^/L) compared to the *ETV6::RUNX1*‐positive patients (P < 0.0001 and P < 0.0001, respectively; Table 1). At a frequency of 12%, Down syndrome was significantly more prevalent among *ETV6::RUNX1*‐like patients (P = 0.0002).

Treatment of *ETV6::RUNX1*‐like patients varied (Supporting Information S2: Table 6), but 87/95 patients were treated using protocols with MRD‐based risk‐stratification. According to PCR‐based MRD monitoring, *ETV6::RUNX1*‐like patients had significantly inferior response to induction treatment compared to the *ETV6::RUNX1*‐positive group (66% vs. 44% detectable MRD at the end of induction [EOI], P = 0.0014). The proportion of EOI‐MRD‐positive cases in the BFM subgroup was comparable to the total *ETV6::RUNX1*‐like group. The proportion of patients with MRD ≥ 1E‐3 at the EOI was only 1.7% of patients with *ETV6::RUNX1*‐positive ALL, but 14% within the *ETV6::RUNX1*‐like group (P = 0.0002). Significantly larger proportion of patients with *ETV6::RUNX1*‐like compared to *ETV6::RUNX1*‐positive ALL were stratified into high and medium risk arms of BFM protocols (74% vs. 48%, P = 0.0016).

*ETV6::RUNX1*‐like patients had a significantly worse event‐free survival (EFS) compared to *ETV6::RUNX1*‐positive patients, while their OS was comparable: 5y‐EFS 77% versus 93% (P < 0.0001), 5y‐OS 97% versus 97% (Figure 3A–B). Similar results were obtained when limiting the analysis to the BFM subgroup of *ETV6::RUNX1*‐like cases (Figure 3C–D) or to the subgroup of *ETV6::RUNX1*‐positive patients with the longest follow‐up (to prevent a possible bias by late relapses; Supporting Information S1: Figure 5).

We analyzed the impact of demographic, clinical, and genetic factors on the outcome of *ETV6::RUNX1*‐like BCP‐ALL (Supporting Information S1: Table 7). According to univariate analyses, age at diagnosis, WBC, NCI risk, MRD at EOI, *IKZF1* deletion, *IKZF1*plus genotype, *CRLF2*r, and *JAK2* mutations had significant prognostic impact for EFS, while age at diagnosis, NCI risk, Down syndrome, *CRLF2*r, and *JAK2* mutations had significant prognostic impact for OS (Table 2 and Supporting Information S1: Figure 6). In multivariate analyses, NCI risk, EOI MRD, *CRLF2*r, *JAK2* mutations, and *IKZF1* deletion retained predictive values for EFS, while Down syndrome, age, and NCI risk remained predictive for OS (Table 3).

**Table 2:** Prognostic impact of selected factors on the outcome of pediatric *ETV6*::*RUNX1*‐like BCP‐ALL analyzed using the univariate Cox model.

Variable	*Gr 1 n (%)*	*Gr 2 n (%)*	*NA n*	Relapse‐free survival (97 children)			Event‐free survival (97 children)			Overall survival (97 children)		
				HR	95% CI	P	HR	95% CI	P	HR	95% CI	P
Age (≥10 years vs. <10 years)	*4* (*4%)*	*93* (*96%)*	*0*	1.9	0.25–14.5	NS	5.16	1.5–17.6	0.009	16.74	3.0–92.6	0.001
NCI risk (SR vs. HR)	*25* (*28%)*	*65* (*72%)*	*7*	0.27	0.01–0.72	0.009	0.23	0.09–0.55	0.001	0.12	0.02–0.61	0.01
WBC (<50E9/I vs. ≥50E9/l)	*69* (*77%)*	*21* (*23%)*	*7*	0.28	0.01–0.08	0.01	0.34	0.14–0.87	0.03	0.35	0.08–1.56	NS
Down syndrome (presence vs. absence)	*11* (*12%)*	*83* (*98%)*	*3*	0.89	0.20–3.89	NS	1.6	0.54–4.77	NS	5.99	1.3–27.1	0.02
EOI MRD (<1E−3 or negative vs. ≥1E−3)	*12* (*14%)*	*73* (*86%)*	*12*	0.29	0.0–0.83	0.02	0.26	0.10–0.70	0.008	0.60	0.07–5.37	NS
*IKZF1* deletion (presence vs. absence)	*46* (*52%)*	*43* (*48%)*	*8*	4.7	1.3–16.6	0.02	2.85	1.1–8.2	0.04	1.20	0.27–5.37	NS
*IKZF1*plus genotype (presence vs. absence)	*30* (*34%)*	*59* (*66%)*	*8*	3.2	1.2–8.74	0.02	2.66	1.1–6.6	0.04	3.06	0.67–13.94	NS
*CRLF2*r (presence vs. absence)	*13* (*13%)*	*84* (*87%)*	*0*	4.3	1.6–11.6	0.003	4.76	2.0–11.4	0.0004	11.36	3.3–48.4	0.001
*JAK2* mutation (presence vs. absence)	*5* (*5%)*	*92* (*95%)*	*0*	32.9	8.6–125.7	3.4E−6	21.91	6.9–69.2	1.4E‐7	6.42	1.3–32.0	0.02

Abbreviations: CI, confidence interval; Gr, groups resulting from stratification by variable; HR, hazard ratio; NA, data not available; P, P value; NS, not significant (P ≥ 0.05).

**Table 3:** Prognostic impact of selected factors on the outcome of pediatric *ETV6*::*RUNX1*‐like BCP‐ALL analyzed using a multivariate Cox model.

Tested variables				
Age	≥10 years versus <10 years			
NCI risk	Standard risk (SR) versus high risk (HR)			
WBC	<50E9/L versus ≥50E9/L			
EOI MRD	(<1E−3 or negative) versus ≥1E−3			
Down syndrome, *CRLF2*r, *JAK2* mutation, *IKZF1* deletion, *IKZF1*plus genotype	Presence versus absence			

Results of the Cox proportional hazard model^a^ for event‐free survival				
Tested variables: WBC category + Age category + NCI risk + *CRLF2*r + EOI MRD category + *IKZF1* deletion + *IKZF1*plus genotype				
Cohort: 75 patients, 14 events				
	Hazard ratio	Lower 95% CI	Upper 95% CI	P
NCI standard risk	0.16	0.05	0.52	0.002
*CRLF2*r present	10.40	2.56	42.30	0.001
*IKZF1* deletion present	4.57	1.18	17.66	0.028

Tested variables: Age category + *CRLF2*r + EOI MRD category + *IKZF1* deletion + *IKZF1*plus genotype + *JAK2* mutation				
Cohort: 81 patients, 17 events				
	Hazard ratio	Lower 95% CI	Upper 95% CI	P
*JAK2* mutations	39.74	9.36	168.74	6E−07
EOI MRD < 1E−3 or negative	0.19	0.06	0.59	0.004

Results of the Cox proportional hazard model^a^ for overall survival				
Tested variables: Down syndrome + Age category + NCI risk + *CRLF2*r				
Cohort: 90 patients, 7 events				
	Hazard ratio	Lower 95% CI	Upper 95% CI	P
Down syndrome present	12.75	12.75	12.75	0.023
Age ≥ 10 years	13.82	13.82	13.82	0.025
NCI standard risk	0.07	0.07	0.07	0.027
*CRLF2*r present	4.98	4.98	4.98	0.076

Abbreviations: CI, confidence interval; P, P value.

a Best model based on Akaike Information Criterion.

## DISCUSSION

The *ETV6::RUNX1*‐like ALL is defined by an *ETV6::RUNX1* gene expression signature and the absence of known subtype‐defining genetic aberrations.^1^, ^6^ Unlike genetic‐based classification, which allows analysis of individual samples, gene expression‐based classification requires comparison to a reference cohort. Although used increasingly, this analysis may not be widely available for diagnostic purposes. Recently, several GEP‐based subtype classifiers have been developed using data from large, well‐annotated reference ALL cohorts, enabling classification of individual ALL cases.^19^, ^20^, ^21^ GEP data of CZE patients were included in the validation of the ALLCatchR classifier (“CLIP hold‐out cohort”),^21^ and all 16 *ETV6*::*RUNX1*‐like cases were classified correctly. Thus, it can be assumed that although co‐clustering with *ETV6::RUNX1* cases remains the only legitimate way to identify *ETV6::RUNX1*‐like samples, the vast majority of them will be classified correctly even by high‐quality classifiers.

Immunophenotypic or genetic profiles can serve as surrogate pointers to *ETV6::RUNX1*‐like subtype classification. Here, we have reinforced our previous observation that CD27^pos^/CD44^low‐neg^ immunophenotype strongly correlates with *ETV6::RUNX1*‐positive/‐like subtype, which may serve as a rapid and economical pre‐screening method to narrow down candidates for GEP. However, with 93% sensitivity as achieved in this study, a minority of ETV6::RUNX1‐like cases will still be missed by this approach.

Similar to immunophenotyping, genetic profiling cannot fully substitute for GEP in *ETV6::RUNX1*‐like subtype classification. Our study confirmed the high frequency of *ETV6* and *IKZF1* aberrations in *ETV6::RUNX1*‐like ALL, justifying pre‐screening for these aberrations prior to WTS in settings where comprehensive sequencing is not feasible. However, their use as diagnostic markers is limited by the lack of universality and specificity, even when focusing on a subset of BCP‐ALL without any known genetic subtype‐defining aberrations. Lesions involving *IKZF1* were absent in more than a third of our cases and in an even larger proportion of *ETV6::RUNX1*‐like ALL in previous studies.^1^, ^4^, ^16^ Lesions of *ETV6* were almost universally present in *ETV6::RUNX1*‐like ALL in our study (96% of cases), while they were slightly less frequent in previous reports.^1^, ^3^, ^4^, ^16^ However, *ETV6* aberrations, particularly deletions, were far from exclusive to *ETV6::RUNX1*‐like ALL. Moreover, *ETV6* fusions with kinase genes occur in *BCR::ABL1*‐like ALL, and *PAX5*::*ETV6* in the *PAX5*alt subtype.^1^ Other *ETV6* fusions, alone or combined with additional lesions resulting in biallelic *ETV6* disruption or with *IKZF1* lesions, may be more specific for *ETV6::RUNX1*‐like ALL, but these are relatively rare. All these limitations must be considered when comparing findings from studies describing “genetically defined *ETV6::RUNX1*‐like” ALL^7^, ^8^ with those utilizing a genuine gene‐expression‐based *ETV6::RUNX1*‐like classification, as the former are likely to comprise 40%–50% of cases lacking the specific GEP, while some cases may be missed.

Although not occurring exclusively in *ETV6::RUNX1*‐like ALL, *ETV6* inactivation, resulting from a variety of genetic mechanisms, remains the top candidate driver of the specific gene expression signature. In our present study (and also in previously published data^1^), a minor subset of *ETV6::RUNX1*‐like ALL had kinase/cytokine receptor gene fusions. Fusions involving *JAK2* and *PDGFRB* occurred in 3 cases in the present study, fusion involving *STYK1* kinase in a single case in the study by Brady et al.,^1^ and *CRLF2*r was found in 14% of *ETV6::RUNX1*‐like ALL in the present study and in 5% in Brady et al. Such fusions are typically associated with *BCR::ABL1*‐like GEP signature. The *ETV6*::*RUNX1‐*like GEP signature may be associated with *ETV6* lesions, which are generally rare in ALL with kinase/cytokine receptor gene fusions,^1^ but were found in all *ETV6::RUNX1*‐like cases with these fusions in our study. Yet again, *ETV6* inactivation in ALL with *CRLF2*r or *ETV6::ABL1* fusion does not necessarily result in *ETV6::RUNX1*‐like GEP. Similarly, only a minority of ALL with *IGH::IGF2BP1* appear to display the *ETV6::RUNX1*‐like phenotype, as demonstrated by a recent study showing a lack of a specific gene expression signature for *IGH::IGF2BP1*‐positive ALL, with some of the cases clustering into or near the *ETV6::RUNX1*‐positive/‐like ALL.^16^ Since *IGF2BP1* is overexpressed in *ETV6::RUNX1*‐positive ALL and contributes to the oncogenic role of *ETV6::RUNX1,* ^22^, ^23^ the biological impact of *IGH::IGF2BP1* and *ETV6::RUNX1* certainly overlaps. However, *IGF2BP1* overexpression mediated by the *IGH* enhancer seems to be insufficient to drive the *ETV6::RUNX1*‐like GEP, and a broader disruption of the *ETV6* transcriptional program is probably required, as suggested by the *ETV6* lesion present in both *IGH::IGF2BP1*‐positive *ETV6::RUNX1*‐like patients. Only four patients with *ETV6::RUNX1*‐like had no detectable *ETV6* lesion. *TCF3::FLI1*, *FUS::ERG*, and *EWSR1::FLI1* fusions were found individually in three of these patients, and rare patients with *TCF3::FLI1* or *FUS::ERG*‐positive *ETV6::RUNX1*‐like ALL without detectable *ETV6* gene disruption are reported in a previously published study.^1^ Like *ETV6*, both *FLI1* and *ERG* belong to the same family of Ets transcription factors and play important roles in hematopoiesis. In addition to ALL, *FUS::ERG* and *EWSR1::FLI1* were described in other malignancies, including Ewing sarcoma, where *EWSR1::FLI1* was shown to bind to *ETV6* targets and to antagonize *ETV6*‐mediated transcriptional repression.^24^, ^25^ We speculate that the abovementioned fusions containing *FLI1* or *ERG* DNA‐binding domains may result in an *ETV6::RUNX1*‐like gene expression profile via a similar mechanism not requiring genetic disruption of *ETV6*.

By definition, GEP is necessary for the identification of *ETV6::RUNX1*‐like ALL, but its limited availability in many diagnostic centers raises the question of whether *ETV6::RUNX1*‐like diagnostics is clinically needed. As mentioned earlier, the reported excellent outcome of genetically defined “*ETV6::RUNX1*‐like” ALL^7^, ^8^ must be interpreted with caution. Our data do not indicate such an excellent prognosis of *ETV6::RUNX1*‐like patients, but confirm, as previously proposed, an intermediate outcome.^1^, ^5^, ^6^ The relapse rate of *ETV6::RUNX1*‐like ALL was significantly higher than *ETV6::RUNX1*‐positive ALL. Children with *ETV6::RUNX1*‐like ALL were younger than those with *ETV6::RUNX1*‐positive ALL, but had higher initial WBC counts, and showed worse response to initial treatment. We showed that high WBC, high EOI MRD, and older age (although infrequent) were associated with worse outcomes in *ETV6::RUNX1*‐like ALL. These well‐established high‐risk factors are included in risk stratification algorithms across various modern treatment protocols.^26^ Therefore, patients at increased risk of relapse are likely to be stratified to more intensive treatment regardless of their *ETV6::RUNX1*‐like status. The same applies to *IKZF1* deletions,^27^ which are rare in *ETV6::RUNX1*‐positive^28^ but occurred in half of *ETV6::RUNX1*‐like ALL cases. *CRLF2*r and *JAK2* mutations, significantly more frequent in *ETV6::RUNX1*‐like than in *ETV6::RUNX1*‐positive ALL (15% and 5% of patients, respectively), were also identified as high‐risk genetic factors in our study. Of note, *JAK2* mutations were present only in patients with *CRLF2*r, all of whom relapsed or died. Results from previous studies evaluating the prognostic value of *CRLF2*r or *JAK2* mutations in BCP‐ALL were inconsistent;^29^, ^30^ however, the poor prognosis of *CRLF2*r combined with *JAK2* mutations has also been reported in patients with *BCR::ABL1*‐like ALL.^31^ Therefore, a refined subtype classification including GEP‐defined subtypes seems essential to further elucidate the prognostic impact of *CRLF2*r and/or *JAK2* mutations.

The OS of *ETV6::RUNX1*‐like was comparable to *ETV6::RUNX1*‐positive ALL. Yet, we found predictors for OS in *ETV6::RUNX1*‐like ALL, including Down syndrome. The frequency of Down syndrome was surprisingly high in our cohort (13%)—if confirmed in further studies, this would make *ETV6::RUNX1*‐like the ALL subtype with the highest incidence of Down syndrome. Consistent with the known higher susceptibility of Down syndrome patients to chemotherapy toxicity,^32^ the cause of death was therapy‐related in two of the three deceased patients.

In summary, we demonstrate a diverse, *ETV6*‐centered, genomic background of *ETV6::RUNX1*‐like ALL. We show that immunophenotyping and genetic profiling can assist in identifying *ETV6::RUNX1*‐like ALL, but gene expression profiling remains essential for accurate classification of this subtype. We confirm an intermediate prognosis of *ETV6::RUNX1*‐like ALL, even in protocols utilizing MRD‐based risk stratification. Importantly, we show here for the first time that age, initial WBC count (or NCI risk), MRD at EOI, and *IKZF1* gene status have prognostic impact in *ETV6::RUNX1*‐like ALL, and thus their use in risk‐evaluation algorithms is beneficial for patients with this ALL subtype. With respect to this observation, we conclude that *ETV6::RUNX1*‐like patients should not be considered in treatment reduction studies, but there is probably no urgent clinical need to incorporate *ETV6::RUNX1*‐like assessment into routine diagnostics on modern protocols. However, patients with *CRLF2*r combined with *JAK2* mutations, who seem to have a poor prognosis within *ETV6::RUNX1*‐like ALL, might be considered for targeted therapy with JAK/STAT inhibitors. However, larger studies are needed to definitively validate these findings.

## AUTHOR CONTRIBUTIONS

**Marketa Zaliova**: Conceptualization; investigation; writing—original draft; methodology; formal analysis; data curation; project administration; writing—review and editing; resources. **Dagmar Schinnerl**: Investigation; writing—review and editing; methodology; formal analysis; resources; data curation. **Judith M. Boer**: Investigation; methodology; writing—review and editing; formal analysis; resources; data curation. **Aurélie Caye‐Eude**: Investigation; methodology; writing—review and editing; formal analysis; resources; data curation. **Jacqueline Rehn**: Investigation; methodology; writing—review and editing; formal analysis; resources; data curation. **Claire Schwab**: Investigation; methodology; writing—review and editing; formal analysis; resources; data curation. **Chloé Arfeuille**: Writing—review and editing; resources; investigation. **Andishe Attarbaschi**: Writing—review and editing; resources; investigation. **Anke Katharina Bergmann**: Writing—review and editing; resources; investigation. **Karel Fiser**: Formal analysis; writing—review and editing; resources; methodology; investigation. **Hester A. de Groot‐Kruseman**: Writing—review and editing; resources; investigation. **Sabrina Haslinger**: Writing—review and editing; resources; investigation. **Andrea Inthal**: Writing—review and editing; resources; investigation. **Iveta Janotova**: Writing—review and editing; resources; investigation. **Lennart Lenk**: Writing—review and editing; Resources; Investigation. **Margarita Maurer‐Granofszky**: Writing—review and editing; resources; investigation. **Karin Nebral**: Writing—review and editing; resources; investigation. **Fiona Poyer**: Writing—review and editing; resources; investigation. **Lucie Sramkova**: Writing—review and editing; resources; investigation. **Jan Stary**: Writing—review and editing; resources; investigation. **Marion Strullu**: Writing—review and editing; resources; investigation. **Jan Stuchly**: Writing—review and editing; formal analysis; resources; methodology; investigation. **Rosemary Sutton**: Writing—review and editing; resources; investigation. **Martina Vaskova**: Writing—review and editing; investigation; resources; methodology. **Lucie Winkowska**: Writing—review and editing; investigation; methodology; resources. **Gunnar Cario**: Writing—review and editing; supervision; resources; investigation. **Hélène Cavé**: Writing—review and editing; supervision; resources; investigation. **Monique L. den Boer**: Writing—review and editing; resources; supervision; investigation. **Christine Harrison**: Writing—review and editing; supervision; resources; investigation. **Sabine Strehl**: Writing—review and editing; supervision; resources; investigation. **Deborah White**: Writing—review and editing; supervision; resources; investigation. **Jan Trka**: Writing—review and editing; conceptualization; supervision; resources; investigation. **Jan Zuna**: Conceptualization; writing—original draft; supervision; data curation; resources; formal analysis; writing—review and editing; project administration; investigation.

## CONFLICT OF INTEREST STATEMENT

Gunnar Cario: research grants—Amgen, Clinigen, Jazz, Servier; consulting fees—Amgen; advisory boards—Amgen, Jazz; travel grants/honoraria—Amgen, Clinigen, Jazz. The remaining authors have no conflicts of interest to declare.

## FUNDING

The study was co‐funded by the EU and the State Budget of Czechia OP JAC, project SALVAGE, No. CZ.02.01.01/00/22_008/0004 644; Blood Cancer, UK (CS and CH); NHMRC, MRFF and CCSA (JR and DW); Fondation ARC (www.fondation-arc.org) (AC‐E and HC); grant of the Anniversary Fund of the Oesterreichische National Bank (OeNB 18 281) (SS). The authors would like to thank to VIVO Biobank for UK samples, to Center for Biological Resources (CRBcancer; BB‐0033‐00 076) of the Robert Debré Hospital, and to Next Generation Sequencing Facility at Vienna BioCenter Core Facilities (VBCF), a member of the Vienna BioCenter (VBC), Austria, for RNA‐seq.

## Supporting information

ETV6‐RUNX1‐like Supplementary Tables and Figures HemaSphere rev2.

ST HemaSphere rev.

## Data Availability

The data that support the findings of this study are available from the corresponding author upon reasonable request. Demographic, genetic, and other data may be found in data supplements available with the online version of this article. For data not found there, please contact the corresponding author.
