# Supplementary material for: Biological and clinical characteristics of ETV6::RUNX1‐like ALL
Source: Hemasphere. 2026 Apr 17;10(4):e70342. doi: 10.1002/hem3.70342 (PMC13088304; doi:10.1002/hem3.70342)
Supplement: Supplementary file 1 — ETV6‐RUNX1‐like Supplementary Tables and Figures HemaSphere rev2. [file HEM3-10-e70342-s001.docx]

**Biological and clinical characteristics of ETV6::RUNX1-like ALL**

**SUPPLEMENTARY TABLES AND FIGURES**

**Supplementary Table 1.** **WTS cohorts stratified by subtype-defining and additional genetic lesions**

|  |  | AUS | | AUT | | CZE^b^ | | | | | | FRA | | NLD | |  | *B-other ETV6del^c^* | |
| --- | --- | --- | --- | --- | --- | --- | --- | --- | --- | --- | --- | --- | --- | --- | --- | --- | --- | --- |
|  |  |  |  |  |  | CZE | | GER | | UK | |  |  |  |  |  |  |  |
|  |  |  |  |  |  |  |  |  |  |  |  |  |  |  |  |  |  |  |
| **BCP-ALL - total number** | | **567^a^** | | **317** | | **323** | | **43** | | **28** | | **244** | | **505** | |  | ***84*** | |
|  |  |  |  |  |  |  |  |  |  |  |  |  |  |  |  |  |  |  |
| BCP-ALL subtypes | | n | % | n | % | n | % | n | % | n | % | n | % | n | % |  | *n* | *%* |
| *ETV6*::*RUNX1* | | 45 | 7.9 | 21 | 6.6 | 15 | 4.6 | _ | _ | _ | _ | 14 | 5.7 | 87 | 17.2 |  | *_* | *_* |
| *BCR*::*ABL1* | | 106 | 18.7 | 6 | 1.9 | 3 | 0.9 | _ | _ | _ | _ | 2 | 0.8 | 41 | 8.1 |  | *_* | *_* |
| *TCF3*::*PBX1* | | 17 | 3.0 | 9 | 2.8 | 1 | 0.3 | _ | _ | _ | _ | 2 | 0.8 | 15 | 3.0 |  | *_* | *_* |
| *TCF3*::*HLF* | | 2 | 0.4 | 0 | 0.0 | 0 | 0.0 | _ | _ | _ | _ | 2 | 0.8 | 1 | 0.2 |  | *_* | *_* |
| *KMT2A*r | | 27 | 4.8 | 6 | 1.9 | 12 | 3.7 | _ | _ | _ | _ | 11 | 4.5 | 20 | 4.0 |  | *_* | *_* |
| Hyperdiploidy (> 50 chr) | | 52 | 9.2 | 22 | 6.9 | 67 | 20.7 | 1^f^ | 2.0 | _ | _ | 52 | 21.3 | 139 | 27.5 |  | 1^f^ | *_* |
| Hypodiloidy (< 44 chr) | | 22 | 3.9 | 8 | 2.5 | 4 | 1.2 | _ | _ | _ | _ | 2 | 0.8 | 6 | 1.2 |  | *_* | *_* |
| iAMP21 | | 5 | 0.9 | 6 | 1.9 | 8 | 2.5 | 0 | 0.0 | 0 | 0.0 | 3 | 1.2 | 29 | 5.7 |  | *2* | *2.3* |
| *DUX4*r | | 36 | 6.3 | 62 | 19.6 | 63 | 19.5 | 2 | 4.7 | 3 | 10.7 | 27 | 11.1 | 16 | 3.2 |  | *9* | *10.5* |
| *ZNF384*r/*ZNF362*r/ | | 18 | 3.2 | 14 | 4.4 | 15 | 4.6 | 7 | 16.3 | 7 | 25.0 | 5 | 2.0 | 4 | 0.8 |  | *14* | *16.3* |
| *MEF2D*r | | 3 | 0.5 | 5 | 1.6 | 6 | 1.9 | 1 | 2.3 | 1 | 3.6 | 5 | 2.0 | 3 | 0.6 |  | *3* | *3.5* |
| *NUTM1*r | | 1 | 0.2 | 5 | 1.6 | 5 | 1.5 | 0 | 0.0 | 0 | 0.0 | 10 | 4.1 | 4 | 0.8 |  | *0* | *0.0* |
| *PAX5* p.(P80R) | | 16 | 2.8 | 7 | 2.2 | 8 | 2.5 | 1 | 2.3 | 1 | 3.6 | 0 | 0.0 | 3 | 0.6 |  | *2* | *2.3* |
| *IKZF1* p.(N159Y) | | 4 | 0.7 | 2 | 0.6 | 3 | 0.9 | 0 | 0.0 | 1 | 3.6 | 4 | 1.6 | 0 | 0.0 |  | *2* | *2.3* |
| *UBTF*::*ATXN7L3* | | 4 | 0.7 | 3 | 0.9 | 1 | 0.3 | 0 | 0.0 | 0 | 0.0 | 0 | 0.0 | 0 | 0.0 |  | *0* | *0.0* |
| *BCL2*r/*MYC*r | | 3 | 0.5 | 1 | 0.3 | 0 | 0.0 | 0 | 0.0 | 0 | 0.0 | 2 | 0.8 | 0 | 0.0 |  | *0* | *0.0* |
| *ZEB2* p.(H1038R) / *IGH*::*CEBP*x | | 5 | 0.9 | 2 | 0.6 | 5 | 1.5 | 0 | 0.0 | 0 | 0.0 | 6 | 2.5 | 6 | 1.2 |  | *0* | *0.0* |
| None of above | *PAX5 fusion* | 9 | 1.6 | 15 | 4.7 | 20 | 6.2 | 4 | 9.3 | 3 | 10.7 | 26 | 10.7 | 2 | 0.4 |  | *8* | *9.3* |
|  | *PAX5^AMP^* | 1 | 0.2 | 13 | 4.1 | 5 | 1.5 | 0 | 0.0 | 0 | 0.0 | 5 | 2.0 | 1 | 0.2 |  | *0* | *0.0* |
|  | *CRLF2r* | 67 | 11.8 | 12 | 3.8 | 19 | 5.9 | 3 | 7.0 | 2 | 3.6 | 19 | 7.8 | 55 | 10.9 |  | *6* | *7.0* |
|  | *kinase fusion^d^* | 39 | 6.9 | 19 | 6.0 | 3 | 1.2 | 1 | 0.0 | 1 | 3.6 | 10 | 4.1 | 11 | 2.2 |  | *2* | *2.3* |
|  | *None of above* | 85 | 15.0 | 79 | 24.9 | 60 | 18.3 | 23 | 55.8 | 9 | 35.7 | 37 | 15.2 | 62 | 12.3 |  | *35* | *43.0* |
|  |  |  |  |  |  |  |  |  |  |  |  |  |  |  |  |  |  |  |
| *classified as ETV6::RUNX1-like* | | *12* |  | *17* |  | *16* |  | *16* |  | *7* |  | *15* |  | *18* |  |  | *24* |  |
|  | | | | | | | | | | | | | | | | | | |
| a, AUS cohort included 249 chidren and 318 adults; b, WTS of patients from CZE, GER and UK was perfromed in CZE; c, subcohort of CZE, GER and UK patients selected for presence of *ETV6* deletion (*ETV6*del) and absence of routinely screened genetic subtype-defining aberrations analyzed in CZE; d, fusions involving kinase (e.g. *ABL1*, *ABL2*, *JAK2*) or cytokine-receptor gene (e.g. *PDGFRB*, *EPOR*) excluding *BCR*::*ABL1*; f, re-classified as Hyperdiploid based on DNA index 1.1 an co-clustering with hyperdiploid BCP-ALL.  WTS, whole transcriptome sequencing; AUS, Australia; AUT, Austria; CZE, Czech Republic; GER, Germany; UK, United Kingdom; FRA, France; NLD, Netherlandes; chr, chromosome | | | | | | | | | | | | | | | | | | |

**Supplementary Tables 2-6 are provided as Excel spreadsheets:**

Supplementary Table 2. Panels of genes for targeted sequencing.

Supplementary Table 3. Gene fusions and other structural rearrangements identified by WTS.

Supplementary Table 4. SNV/indels identified by WTS, WES or targeted NGS.

Supplementary Table 5. Copy number aberrations and copy neutral losses of heterozygosity identified by SNP array, MLPA and targeted NGS.

Supplementary Table 6. Demographic, clinical, biological and genetic features of 101 patients with *ETV6*::*RUNX1*-like BCP-ALL.

**Supplementary Table 7. Survival of children with *ETV6::RUNX1*-like BCP-ALL (n = 97) – univariate Cox model.**

**Supplementary Figure 1. Description of cohorts of patients diagnosed and treated in the Czech Republic and included in present study**

|  |  | |  | |  |  | |  | |  | |  |
| --- | --- | --- | --- | --- | --- | --- | --- | --- | --- | --- | --- | --- |
| 706 patients BCP-ALL (XII/2010 - X/2021) | 705 patients with BCP-ALL classified into subtypes^a^ | | 351 (50%) | | Positive for hyperdiploidy/*KMT2A*r/*TCF3*::*PBX1*/*BCR*::*ABL1*/hypodiploidy | | | | | | | |
|  |  |  | 177 (25%) | | *ETV6*::*RUNX1* positive^b^ | | | | | | | |
|  |  |  | 177 (25%) | | Negative for  hyperdiploidy *KMT2A*r *TCF3*::*PBX1* *BCR*::*ABL1* hypodiploidy *ETV6*::*RUNX1* | 13 | ETV6::RUNX1-like | | 12 | | GEP study YES^d^, SNP array study YES^e^ | |
|  |  |  |  |  |  |  |  |  | 1 | | GEP study YES^d^, SNP array study NO | |
|  |  |  |  |  |  | 164 | other subtypes | | 148 | | GEP study YES^d^, SNP array study YES^e^ | |
|  |  |  |  |  |  |  |  |  | 9 | | GEP study YES^d^, SNP array study NO | |
|  |  |  |  |  |  |  |  |  | 7 | | GEP study NO, SNP array study YES^e^ | |
|  | 1 patients with BCP-ALL negative for hyperdiploidy/*KMT2A*r/*TCF3*::*PBX1*/*BCR*::*ABL1*/hypodiploidy, not further classified | | | | | | | | | | | |
|  |  | | |  |  |  | |  | |  | |  |
| ^a^ Cohort used to estimate frequency of *ETV6*::*RUNX1*-like BCP-ALL | | | | | | | | | | | | |
|  | | | | | | | | | | | | |
| ^b^ Cohort (n=177) used in analyses comparing demographic and diagnostic parametres and survival between *ETV6*::*RUNX1*-like and *ETV6*::*RUNX1*-positive BCP-ALL. | | | | | | | | | | | | |
|  | | | | | | | | | | | | |
| ^d^ Cohort (n=170) included in the gene expression based *ETV6*::*RUNX1*-like classification in present study. Cohort represents 96% of 178 patients consecutively diagnosed with BCP-ALL negative for hyperdiploidy/*KMT2Ar*/*TCF3*::*PBX1*/*BCR*::*ABL1*/hypodiploidy. Out of 8 patients not included in the present study, subtyped-defining genetic aberrations (precluding classification as *ETV6*::*RUNX1*-like) were found in 7 patients, subtype was incompletely specified in 1 patient. | | | | | | | | | | | | |
|  | |  | |  |  |  | |  | |  | |  |
| ^e^ Cohort of 167 patients with available SNP array data representing 94% of all pateints consecutively diagnosed with BCP-ALL negative for routinely screened genetic aberrations was used to study association of *ETV6* and *IKZF1* aberrations with *ETV6*::*RUNX1*-like phenotype. | | | | | | | | | | | | |
| GEP – gene expression profiling | | | | | | | | | | | | |

**Supplementary Figure 2. *ETV6*-*RUNX1*-like classification**

BCP-ALL were classified as *ETV6::RUNX1*-like according to the algorithm published by Brady et al. (Nature Genetics 2022; doi: 10.1038/s41588-022-01159-z). BCP-ALL harboring subtype-defining genetic aberrations (*ETV6::RUNX1*, *BCR::ABL1*, *TCF3::PBX1*, *TCF3::HLF*, *KMT2A*r, high hyperdiploidy, hypodiploidy, iAMP21, *DUX4*r, *ZNF384*r, *MEF2D*r, *NUTM1*r, *PAX5* P80R, *IKZF1* N159Y, *UBTF::ATXN7L3*, *BCL2*r, *MYC*r, *ZEB2* H1038R, *IGH::CEBP*) were classified into 17 corresponding genetically defined subtypes. The remaining BCP-ALL cases (lacking all subtype-defining genetic aberrations listed above) were further classified based on the gene expression profiling. Samples co-clustering with *ETV6::RUNX1-positive* BCP-ALL in unsupervised analysis were classified as *ETV6::RUNX1*-like. Samples not clustering with *ETV6::RUNX1-positive* BCP-ALL (i.e., samples co-clustering with any other genetically defined subtypes such as *BCR::ABL1*, *ZNF384r,* etc., and samples remaining outside of well-defined clusters) were not classified as *ETV6::RUNX1*-like. In acordance with the algorithm published by Brady et al., *ETV6::RUNX1-like* subtype was mutually exclusive with *BCR::ABL1-like* and other GEP-defined subtypes.

Abbreviations: HHD – high hyperdiploidy, r – rearrangement, chr – chromosomes, iAMP – intrachromosomal amplification, GEP – gene expression profile.

**Supplementary Figure 3. *ETV6* gene rearrangements**


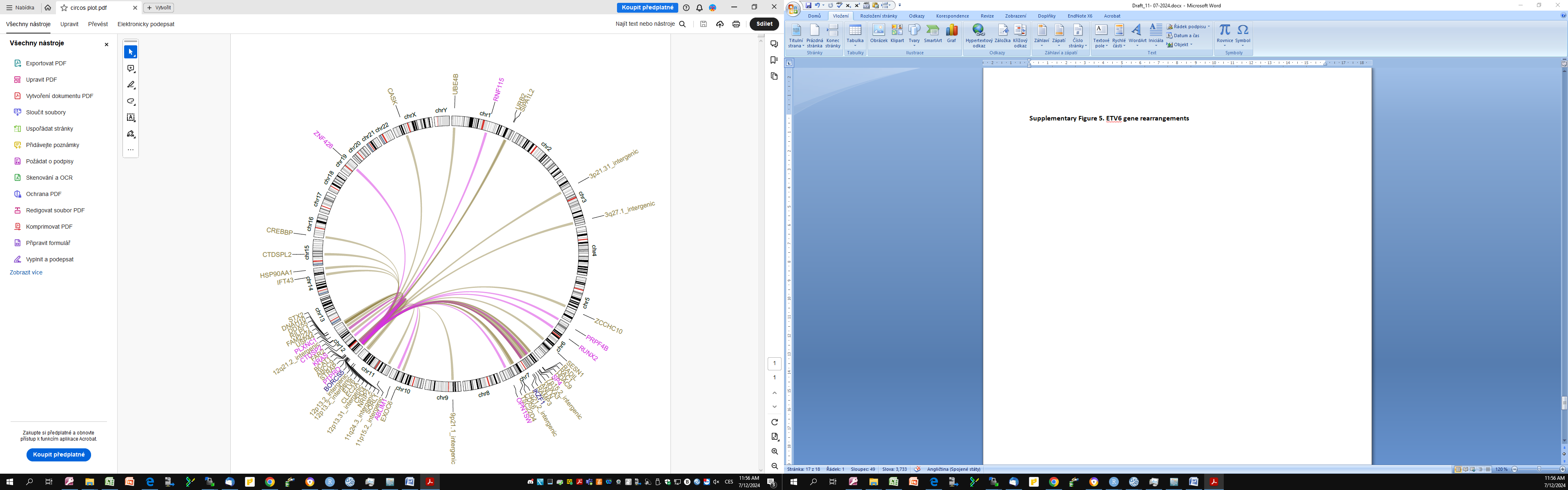


The Circos plot was constructed using shinyCircos- v2.0 (<https://venyao.xyz/shinyCircos/>) and illustrates 67 rearrangements of *ETV6* found in 47 patients. An additional rearrangement of *ETV6* with unknown gene/region was found by FISH in additional patient and is not included in figure. In-frame fusions are highlighted in magenta, while other rearrangements are shown in light brown. *BORCS5* and *IKZF1*, highlighted in dark navy color, were recurrent *ETV6* partners and occurred in both in-frame and out-of-frame fusions. The only other recurrent partners were *FAR2* and *DNAH10* genes, located on chromosome 12.

**Supplementary Figure 4. Frequency of *ETV6*::*RUNX1*-like subtype and of selected genetic lesions in a consecutive “B-other” cohort.**


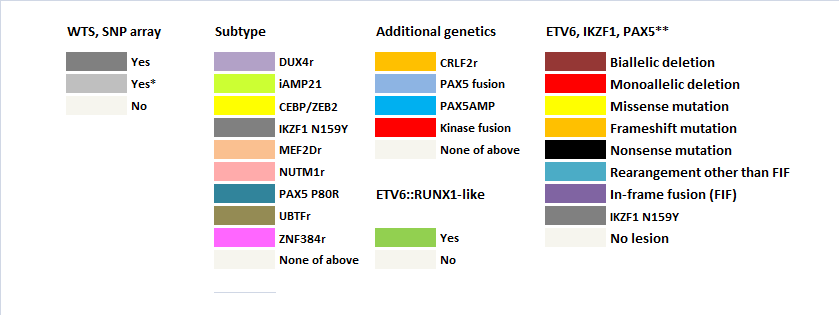


The scheme illustrates the classification and genetic features of 178 patients representing all children consecutively diagnosed with BCP-ALL who were negative for *ETV6*::*RUNX1*, *BCR*::*ABL1*, *TCF3*::*PBX1*, *KMT2A*r, hyperdiploidy and hypodiploidy in the Czech Republic between December 2010 and October 2021. SNP array data were available in 167 patients. WTS refers to whole transcriptome sequencing. "Yes*" indicates that WTS was performed to identify subtype-defining genetic lesions, but the WTS data were not used for gene expression-based classification in the present study. "**" denotes that only *PAX5* deletions (not fusions or SNV/indels) are included in the scheme.

**Supplementary Figure 5. Outcome of pediatric patients with *ETV6*::*RUNX1*-like patients versus 123 *ETV6*::*RUNX1*-positive patients diagnosed and treated in the Czech Republic from December 2010 to September 2017**

(A-B) Treatment outcomes of *ETV6*::*RUNX1*-like patients (n=97) and *ETV6*::*RUNX1*-positive (n=123) patients diagnosed and treated in the Czech Republic from December 2010 to September 2017 (i.e. with follow-up > 6 years). (C-D) ETV6::RUNX1-like patients stratified according to the type of treatment protocol (BFM: MRD-guided AIEOP-BFM protocols 2000, 2009 and 2017; nonBFM: other protocols, for details see Supplementary Table 6), compared to *ETV6*::*RUNX1*-positive (n=123) patients. Censoring times are indicated by short vertical lines.

EFS – event free survival, OS – overall survival, y – year.

**Supplementary Figure 6. Outcome of pediatric patients with *ETV6*::*RUNX1*-like BCP-ALL stratified by demographic clinical and genetic variables**

Outcomes of *ETV6*::*RUNX1*-like patients stratified by selected variables. WBC, white blood cell count; NCI, ; HR, high risk; SR standard risk; EOI, end of induction treatment; MRD, minimal residual disease; EFS, event free survival; OS, overall survival.

Censoring times are indicated by short vertical lines.

1. Brady SW, Roberts KG, Gu Z, et al. The genomic landscape of pediatric acute lymphoblastic leukemia. *Nat Genet*. 2022;54(9):1376-1389.
